# Evolution and plasticity of anuran larval development in response to desiccation. A comparative analysis

**DOI:** 10.1002/ece3.2

**Published:** 2011-09

**Authors:** Alex Richter-Boix, Miguel Tejedo, Enrico L Rezende

**Affiliations:** 1 Department of Population Biology and Conservation Biology, Evolutionary Biology Centre (EBC), Uppsala UniversityNorbyvägen 18 D, SE-752 36 Uppsala, Sweden; 2 Department of Evolutionary Ecology, Estación Biológica de Doñana-CSICAvda. Américo Vespucio s/n, E-41092 Sevilla, Spain; 3 Departament de Genètica i de Microbiologia, Grup de Biologia Evolutiva, Universitat Autònoma de Barcelona08193 Bellaterra (Barcelona), Spain

**Keywords:** Anuran tadpoles, developmental plasticity, evolutionary trade-off, life-history theory, phylogenetic analyses

## Abstract

Anurans breed in a variety of aquatic habitats with contrasting levels of desiccation risk, which may result in selection for faster development during larval stages. Previous studies suggest that species in ephemeral ponds reduce their developmental times to minimize desiccation risks, although it is not clear how variation in desiccation risk affects developmental strategies in different species. Employing a comparative phylogenetic approach including data from published and unpublished studies encompassing 62 observations across 30 species, we tested if species breeding in ephemeral ponds (*High risk*) develop faster than those from permanent ponds (*Low risk*) and/or show increased developmental plasticity in response to drying conditions. Our analyses support shorter developmental times in *High risk*, primarily by decreasing body mass at metamorphosis. Plasticity in developmental times was small and did not differ between groups. However, accelerated development in *High risk* species generally resulted in reduced sizes at metamorphosis, while some *Low risk* species were able compensate this effect by increasing mean growth rates. Taken together, our results suggest that plastic responses in species breeding in ephemeral ponds are constrained by a general trade-off between development and growth rates.

## Introduction

One of the primary goals of life history theory is to explain the variation in age and size of organisms at ontogenetic niche transitions (hatching, metamorphosis, maturation), and how potential trade-offs may constrain its plasticity and evolution (Wilbur and Collins 1973; Stearns and Koella 1986; Gotthard and Nylin 1995). For instance, when the developmental time is constrained-for example in amphibian tadpoles developing in desiccating ponds (Newman 1992) or insect larvae during shortening day lengths (Johansson et al. 2001)-optimality models predict that organisms should accelerate their development while increasing growth rates, to minimize juvenile mortality and the costs of decreased size at the life-history transition (Abrams et al. 1996; Strobbe and Stoks 2004). However, a faster development may result in fitness costs at other levels, such as decreased survival during adulthood (Arendt 1997; Nylin and Gotthard 1998; Stoks et al. 2006), hence the adaptive value of this response will depend on the balance between its benefits and costs during larval development and the subsequent stages. It remains consequently unclear how species or populations subject to different selection intensities for accelerated development eventually respond to selection, and how they can minimize the impact of increased developmental rates on overall fitness.

**Figure 1:**
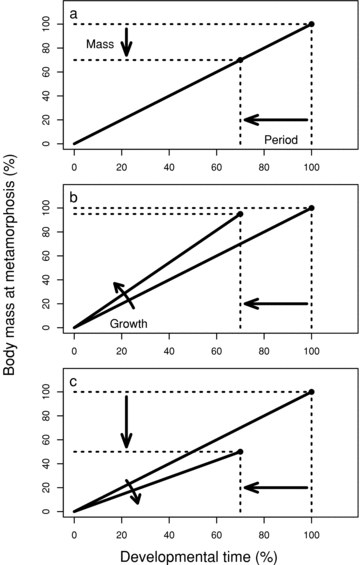
Studying the relationship between developmental rates ( = 1/developmental period), mean growth rates, and body mass at metamorphosis. Compared to the developmental trajectory under nonstressful conditions (i.e., no desiccation) represented by the 1:1 line, accelerating development would result in the following scenarios: (a) if developmental rates and mean growth rates are uncoupled, for example, growth rates may be constant to differing rates of development and thus a decrease in developmental period due to pond drying would be accompanied by a similar reduction in size at metamorphosis; (b) if a minimum viable body size is required to enter metamorphosis or for survival as an adult, mean growth rates should increase to compensate for the accelerated development; and (c) developmental rates would increase at the expense of growth if there is a trade-off between these separate processes. Note that this simplified scheme depicts a linear growth trajectory for illustrative purposes because we work with average growth rates throughout the study, real growth trajectories in anuran tadpoles are by no means linear (see Fig. S1).

Larval amphibians occupy aquatic environments along a wide permanency gradient (Woodward 1983; Richardson 2001; Babbitt et al. 2003), and they are an ideal system for examining the evolution of developmental strategies in response to environmental variability. It is often assumed that species breeding in streams and permanent ponds, which are subjected to low desiccation risk, have longer developmental times than those breeding in temporary and ephemeral ponds (Denver 1997; Wells 2007). If tadpoles must develop faster to minimize desiccation risk, either as an evolutionary response or by means of developmental plasticity, three different scenarios can emerge (Fig. 1). First, developmental period can be modulated by increasing or decreasing body mass at metamorphosis without requiring any change in average growth rate (this scenario is shown in Fig. 1a). Second, tadpoles can grow faster to compensate for the acceleration in development, thus maximizing size at metamorphosis. Such a pattern would suggest that size at metamorphosis has a large effect on fitness and that growth rates are modulated as a correlated response. Third, developmental rates may increase at the expense of growth, and in this scenario tadpoles may speed up metamorphosis at the cost of being smaller at this stage. This would support a trade-off between growth and development, which possibly stems from a limited resource budget that must eventually sustain either cell growth/division or cell differentiation (see Smith-Gill and Berven 1979).

Environmental variability adds another level of complexity because different strategies may be favored depending on the conditions encountered during development. Smaller temporal ponds are expected to appear and disappear within a relatively short period of time, subjecting those species breeding in these habitats to a higher risk of desiccation. Evolutionary responses to increased desiccation risk may involve an overall shift toward faster development, the evolution of plasticity triggered only when ponds are drying (Denver 1997), or both. Intuitively, one would expect that species from more temporary ponds should show an increased plasticity, everything else being equal, because these ponds are intrinsically more variable (Newman 1992; Doughty and Reznick 2004; Wells 2007). Conversely, the opposite pattern should be expected if these species are developing at rates near their maximum physiological capacities (Nunney 1996; Nylin and Gotthard 1998). It is therefore not surprising that empirical studies testing for differences in developmental rates and plasticity between and within species facing contrasting desiccation risks are not conclusive, and have resulted in discordant patterns both at the interspecific and intraspecific level (Brady and Griffiths 2000; Leips et al. 2000; Merilä et al. 2004; Richter-Boix et al. 2006; Lind and Johansson 2007). Disentangling between these two alternatives involve assessing concomitantly (1) whether average developmental rates differ across species facing contrasting risks of desiccation and (2) if these species also exhibit contrasting levels of plasticity in developmental rates around these mean values.

In the present study, we determined whether amphibian anuran species facing higher risks of desiccation have evolved faster developmental rates and increased plasticity than species living in more stable pond environments. We tested these hypotheses with a comparative approach and a large dataset that includes several anuran species belonging to distinct taxonomic groups. Importantly, several comparative studies have shown that evolutionary inferences may change when phylogenetic relations among species are taken into account (e.g., see Gomes et al. 2009 for a recent case study in anurans). We specifically examine the following questions: (1) Do developmental rates, mean growth rates, and mass at metamorphosis differ between species breeding in ponds with different risk of desiccation? Subsequently, we determined if plastic responses showed significant interspecific differences, asking: (2) Is plasticity in developmental rates, mean growth rates, and mass at metamorphosis greater in species with increased risk of desiccation? (3) Are plastic responses in developmental rates and mean growth rates correlated, and do these correlations differ as a function of desiccation risk? Finally, we assess if evolutionary shifts in developmental rates have altered the magnitude and direction of plastic responses across species: (4) Is the variation in mean developmental rates, mean growth rates, and body mass at metamorphosis observed under constant conditions correlated with plasticity in these variables?

## Material and Methods

### Data collection

The literature search was performed in several electronic databases (ISI Web of Science, BasicBIOSIS, BioOne, Biological Abstract, ScienceDirect, Scopus and Scirus), employing the key words “pond desiccation,”“hydroperiod,”“metamorphosis,”“life history,” and “larval period,” and the references of all papers obtained with this search were subsequently reviewed. Data from individual studies were included only if they met the following criteria. First, the study experimentally manipulated water level or water permanency directly by comparing developmental time and size at metamorphosis in constant water-level treatment versus desiccation treatment in the laboratory or mesocosm, or indirectly by comparing permanent versus temporary ponds in the field. When more than one level was available, the extremes were selected to maximize the difference in desiccation risk between treatments. Second, we excluded studies that combined pond drying effects with another variable to avoid confounding effects (in factorial experiments, we compared the control against the treatment where only the drying regime was manipulated). Third, studies that utilized snout vent length as an estimate of metamorphic size were not included, but we did include data from one study that reported body volume (Adams 2000) because this measurement is highly correlated with body mass and can be readily transformed to mass units assuming that density ≈ 1 g/mL. Results from single studies including multiple species or populations were included as independent samples.

We assembled a database comprising 62 independent cases, encompassing a total of 30 species, including the hybrids between *Rana sphenocephala* and *Rana blairi* and the hybrid of *Pelophylax lessonae* and *Pelophylax ridibunda* (*Pelophylax esculenta*), that were considered as ‘ecological’ species (see Data analysis below), from 25 studies in the literature, published between 1989 and 2006, and three unpublished datasets (Table S1). Developmental rates were calculated as 1/developmental period and mean growth rates as body mass/developmental period. This later estimate of mean growth rate was employed for simplicity as a general descriptor of growth rates (see Abrams et al. 1996, pp. 383), because growth trajectories in anurans can be considerably complex (Harris 1999) (see Fig. S1). It must be emphasized that, even though this estimate may be adequate for comparative purposes because it takes into consideration the final outcome of the tadpole's ontogeny (i.e., mass at metamorphosis), it has very little to say about the real shape of the developmental curve during this period. All subsequent statistical analyses have been performed employing developmental rates, though we often discuss results in the context of developmental periods because it is more intuitive (e.g., *Pelodytes ibericus* takes 106 days to reach metamorphic climax which corresponds to a developmental rate of 0.0094 days^−1^, see Table S1).

We operationally assigned species into two categories, according to the typical habitat they employed for breeding, to analyze the evolutionary consequences of developing under contrasting variation of desiccation risks: (1) *High risk*. Species exposed to a high risk of larval mortality by pond desiccation, including ephemeral and temporary ponds that hold water for only a few weeks or months and dry each year, occasionally with several dryings and refills per season. (2) *Low risk*. Species exposed to a lower risk of pond desiccation, basically permanent ponds holding water year-round in most years with rare events of desiccation. Desiccation risk varies more within and between years in ephemeral and temporary ponds than in permanent ponds (Richter-Boix et al. 2006). In most cases, we employed the habitat description provided in the papers to assign species/populations into these categories. When this information was not available, we obtained species breeding habitat from AmphibiaWeb database (http://amphibiaweb.org/) or Global Amphibians Assessments Project database (http://www.globalamphibians.org/) and Lannoo (2005). We also collected information on the experimental venue employed in each study (i.e., laboratory, mesocosm or field conditions).

### Data analysis

Analyses were performed employing phylogentic generalized linear models (see Garland et al. 2005 for a review), with the *ape* package available in R (http://cran.r-project.org/). Because we did not detect any significant effects of different experimental venues in preliminary analyses, values obtained from laboratory, mesocosm, and field experiment were combined in subsequent analyses. Comparisons of developmental strategies between *High* and *Low risk* species were performed employing values measured under constant water-level conditions, because this minimizes the potentially confounding effects of plasticity. We compared developmental rates, mean growth, and body mass with a linear model including desiccation risk as a categorical factor (*High risk* vs. *Low risk*). In these analyses, all variables were log_10_-transformed to meet the assumption of normality. A similar model including developmental time as a covariable was subsequently employed to compare mean growth rates and body mass at metamorphosis controlling for differences in developmental time.

To estimate and compare plastic responses across species, we first accounted for interspecific differences in developmental rates, mean growth rates, and body mass by setting these variables measured under constant conditions to 100%. Subsequently, we expressed estimates obtained in drying conditions as a fraction of this total, and plasticity was estimated as the proportional difference between mean traits under constant and drying conditions (e.g., plasticity in body mass was calculated as 100 × (*mass_d_*−*mass_c_*)/*mass_c_* where *c* and *d* correspond to constant and drying conditions, respectively). To ensure that none of the analyses were affected by variability within treatments, we also estimated plasticity as standardized effect sizes (Hedges' *d* provided by meta-analysis performed with Metawin 2.1, Rosenberg et al. 2000), which was highly correlated with plasticity expressed as percentages (Fig. S2). Results remained qualitatively similar (analyses not shown), therefore we only report results with plasticity estimated as percent changes because their interpretation is more intuitive. Comparisons of developmental plasticity were performed with generalized linear models, as explained above, including desiccation risk as a categorical factor. In addition, we ran different linear models with log_10_-transformed mean traits obtained in constant conditions as the independent variable and plasticity estimates as the dependent variable, to analyze how plasticity varied as a function of mean developmental and mean growth rates and body mass at metamorphosis.

To conduct the phylogenetic analyses, a phylogeny at the family level was constructed following Frost et al. (2006). Importantly, the topology of this backbone phylogeny was congruent with more recent studies based on DNA sequences (Roelants et al. 2007; Wiens 2007). Subsequently, we combined this information with additional detailed within-family phylogenetic assessments for the following families: Scaphiopodidae, Pelobatidae, and Pelodytidae (García-París et al. 2003), Myobatrachidae (Schäuble et al. 2000; Read et al. 2001), Hylidae (Faivovich et al. 2005; Wiens et al. 2006), Bufonidae (Pauly et al. 2004), and Ranidae (Veith et al. 2003; Hillis and Wilcox 2005; Scott 2005). Multiple measurements per species were included as soft polytomies at the tips of the phylogeny, and hybrids were included in a soft polytomy with their respective parental species (Fig. S3). To avoid inflated type I error due to these polytomies (25 in total), we opted for a conservative approach and subtracted one degree of freedom for each unresolved node during hypothesis testing (Purvis and Garland 1993; Garland and Díaz-Uriarte 1999).

We tested if phylogenetic signal-that is, the tendency for related species to resemble each other (Blomberg et al. 2003)-was present for different traits as follows. We ran generalized linear models employing both a star phylogeny (which corresponds to conventional statistical analyses, see Garland et al. 2005) and arbitrary branch lengths according to Pagel (1992), and subsequently determined how well the conventional and the phylogenetic model fitted the phenotypic data employing the Akaike information coefficients (*AIC*) (Burnham and Anderson 2002). The *AIC* criterion is currently a standard tool in model selection, which allows for comparing the goodness of fit of different models while penalizing for increasing the number of estimated parameters (the model with the lowest *AIC* value is considered the best model; Burnham and Anderson 2002). We also computed Akaike weights (*AIC_w_*) to estimate the relative weight of the evidence in favor of each model (Turkheimer et al. 2003), which can be loosely interpreted as the probability of each model being correct given all the models that were tested. These analyses indicate if signal is present in the analyzed dataset and, consequently, which model is more reliable for evolutionary inferences.

## Results

### Evolutionary differences in development

**Figure 2:**
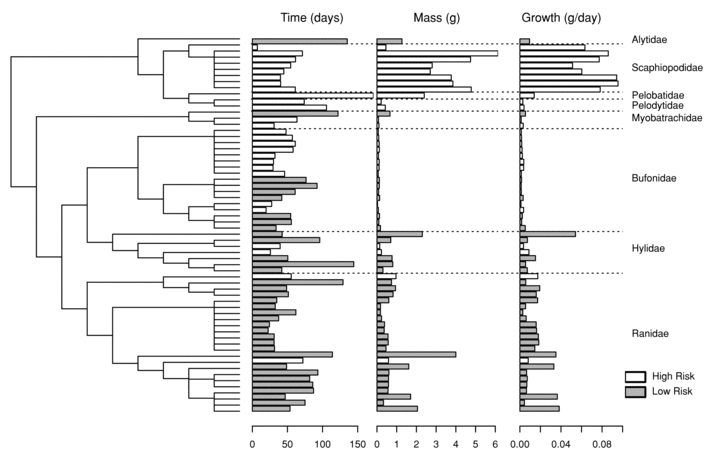
Phylogenetic hypothesis employed in this study, including species/populations developmental times, body mass at metamorphosis, and mean growth rates under constant water level conditions. Species and population identification (see Table S1 and Fig. S3).

**Figure 3:**
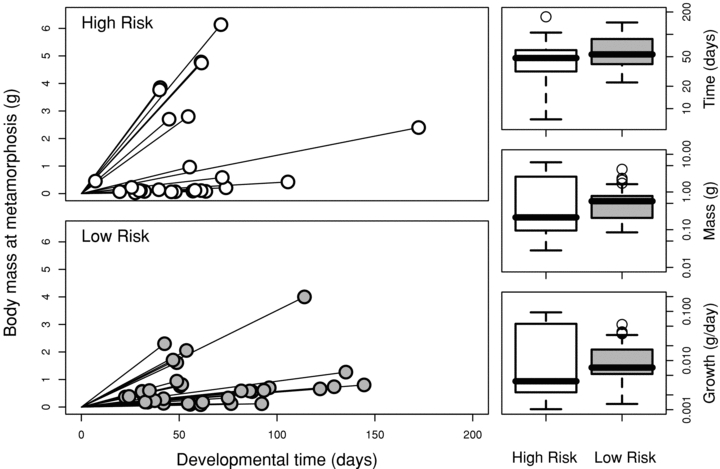
Developmental differences between species breeding in ephemeral (*High risk* in white) and permanent ponds (*Low risk* in gray), where lines schematically show a linear developmental trajectory with a slope corresponding to the average growth rate (see model outlined in Fig. 1 and Fig. S1). Boxplots depict the median, the 25% and 75% CI (box), 5% and 95% CI (error bars), and outliers for developmental period ( = 1/developmental rate), body mass at metamorphosis and mean growth rates obtained under constant water level conditions. Phylogenetic models always resulted in the best fit according to AIC values, and show that developmental rates and body mass at metamorphosis, but not mean growth rates, differ significantly between *High risk* and *Low risk* (Table 1).

Do developmental rates, mean growth rates, and mass at metamorphosis differ between species breeding in ponds with different risk of desiccation? Comparisons between *AIC* and *AIC_w_* show that phylogenetic models comparing developmental rates, mean growth rates, and body mass at metamorphosis had a substantially better fit than conventional analyses (Table 1). This suggests that these traits exhibit high phylogenetic signal (see Fig. 2) and those results from phylogenetic analyses are more reliable. Comparisons controlling for phylogeny indicate that species breeding in ponds with increased desiccation risk have evolved significantly higher developmental rates than their counterparts from more permanent ponds (Table 1 and Fig. 3). This difference seems to be primarily associated with a significantly lower body mass at metamorphosis in species with high risk of desiccation, while no statistical differences in mean growth rates were detected between groups (Table 1). Accordingly, when we control for differences in developmental rates, species from the *High risk* group exhibit significantly lower body mass at metamorphosis and mean growth rates (*P* < 0.01 in both cases), supporting the prediction that these species accelerate their development at the expense of growth (Fig. 1c).

**Table 1:** Evolutionary differences in developmental rates, mean growth rates, and body mass at metamorphosis between species breeding in ponds with *High risk* and *Low risk* of desiccation, measured under constant water-level conditions. Note that 25 degrees of freedom have been subtracted in phylogenetic analyses to account for the soft polytomies (see Methods). Models with the best fit are highlighted in bold

Dependent^1^	Model	Desiccation risk	AIC	AIC_w_^2^
Developmental rates	Conventional	t_60_ = −1.65, P = 0.104	10.37	0.001
	**Phylogenetic**	**t_35_ = −2.91, P = 0.006**	**−3.25**	**0.999**
Mean growth rates	Conventional	t_60_ = 0.047, P = 0.96	114.2	0.00
	**Phylogenetic**	**t_35_ = 1.13, P = 0.266**	**54.7**	**1.00**
Mass at metamorphosis	Conventional	t_60_ = 0.72, P = 0.474	117.7	0.00
	**Phylogenetic**	**t_35_ = 3.39, P = 0.002**	**37.0**	**1.00**

1 Results from general linear models employing log_10_-transformed values (see boxplots Fig. 3).

2 One conventional model and one phylogenetic model were analyzed per dependent variable, hence pairwise *AIC_w_* should add up to one.

### Phenotypic plasticity

Here we test if plasticity in developmental rates, mean growth rates, and mass at metamorphosis are greater in species with increased risk of desiccation. Comparisons between *AIC* values suggest that phylogenetic models provide a better fit to plastic responses for developmental rates and body mass at metamorphosis, whereas conventional analyses are more reliable for mean growth rates (Table S2). Results of conventional and phylogenetic models are qualitatively similar, however, and suggest that plasticity in developmental rates, mean growth rates, and body mass at metamorphosis are similar between *High* and *Low risk* breeders (*P* > 0.170 in all analyses, see Table S2).

**Figure 4:**
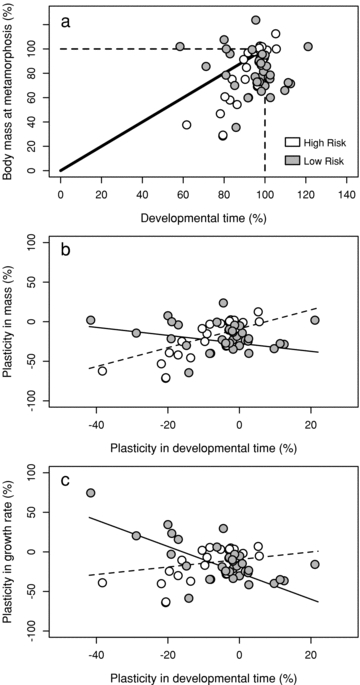
Plastic responses to pond desiccation. (a) Development during drying conditions compared to results observed under constant water level conditions that were set to 100% for comparative purposes (see Methods). (b) The association between plasticity in developmental time and plasticity in body mass at metamorphosis and (c) between plasticity in developmental time and plasticity in mean growth rate (plasticity estimates were calculated as 100 × (*trait_d_*−*trait_c_*)/*trait_c_* where *d* and *c* correspond to drying and constant conditions, respectively). Results of phylogenetic regressions for species breeding in ephemeral ponds (*High risk*) and permanent ponds (*Low risk*) are shown with dashed and solid lines, respectively. The interaction between developmental time plasticity × desiccation risk was significant in analyses of mass and growth plasticity (*P* < 0.0027 in both models), indicating that species from *High risk* and *Low risk* employ different strategies to accelerate their development.

However, species breeding in ephemeral and permanent ponds apparently respond differently to drying conditions: the reduction in developmental time in species from *High risk* was accompanied by a decrease in body mass at metamorphosis (Fig. 4b), whereas the largest reductions in development time in *Low risk* species were observed in species that increased mean growth rates (Fig. 4c). This suggests that a trade-off between developmental rates and mean growth rates exists in species breeding in ephemeral ponds, while several species breeding in permanent ponds seem able to compensate accelerated developmental rates and reach metamorphosis at a relatively constant body mass. These contrasting responses of *Low* and *High risk* species fit well with the expected scenarios proposed in Figure 1b and c, respectively.

### Evolutionary shifts and their association with plasticity

Does the variation in mean estimates measured under constant conditions correlate with plasticity estimates? Phylogenetic generalized linear models suggest that plastic responses in developmental times are relatively constant across species and independent of the absolute length of the development period (*t*_33_ = −0.62, *P* = 0.54). In other words, the reduction in developmental time due to plasticity is a relative constant fraction of developmental times under constant “optimal” conditions, regardless of whether a species takes 20 d or 170 d to develop. Conversely, the relative reduction in body mass at metamorphosis was significantly higher in larger species (*t*_33_ = −2.86, *P* = 0.007, respectively). Thus, larger tadpoles were able to metamorphose at a substantially lower fraction of their mass under constant conditions.

A similar pattern was observed for plasticity in mean growth rates: species growing on average faster showed larger reductions in mean growth rates when exposed to decreasing water levels. This trend was significant according to a conventional linear model (*t*_58_ = −3.84, *P* = 0.0003), which fits the data substantially better (*AIC* = 537.17 and *AIC_w_* = 0.94) than the phylogenetic model (*AIC* = 543.58 and *AIC*_w_ = 0.06). Interestingly, the magnitude of the plastic response in developmental times was not significantly related to variation in either body mass at metamorphosis (*t*_33_ = −0.26, *P* = 0.796) and mean growth rates (*t*_33_ = 0.17, *P* = 0.866), hence the reduction in developmental times seems to be relatively constant across species regardless of their size at metamorphosis and mean growth rates.

## Discussion

To answer the specific issues raised in the introductory section, our results can be succinctly summarized as follows. (1) Species that typically breed in temporary ponds have faster development rates than do species that typically breed in permanent ponds. This evolutionary response is associated with a reduced body size at metamorphosis and lower mean growth rates (Fig. 3). (2) There were no significant differences in plasticity between species breeding in temporary and permanent ponds. Although several species were plastic and capable of decreasing developmental time when exposed to desiccation, this response was not general across taxa (Fig. 4a and Fig. S3). (3) Correlations between developmental rates, mean growth rates, and body mass at metamorphosis differed dramatically between species breeding in temporary and permanent ponds, suggesting that evolution in developmental rates have resulted in changes at the level of plasticity (Fig. 4b and c). (4) Species with either longer larval period or larger body sizes at metamorphosis are not able to decrease their developmental period to a larger extent than other species.

### Evolutionary shifts and plastic responses

Previous studies have suggested that anuran larvae that exhibit shorter mean developmental times in ephemeral ponds with increased risk of desiccation also show reduced body mass at metamorphosis (Denver 1997; Wells 2007). Our analyses show that interspecific variation in developmental rates and body mass at metamorphosis are partly explained by differences in desiccation risk during larval development (Table 1, Fig. 3). Species breeding in ephemeral ponds enter metamorphosis at a comparatively smaller body size than their counterparts breeding in permanent ponds, which seems to explain the significant difference in developmental period observed between groups. If a critical size is necessary to trigger metamorphosis, as proposed by some theoretical models (Wilbur and Collins 1973; Day and Rowe 2002), this would suggest that *High risk* species have evolved lower threshold sizes to reduce developmental time. Morey and Reznick (2004) reported that spadefoot toad species inhabiting ephemeral ponds had the shortest development times and threshold size to enter metamorphosis, and our results suggest that this is a general pattern.

In addition, analyses controlling for differences in developmental rates suggest that species breeding in ephemeral ponds actually grow slower than their counterparts breeding in permanent ponds. This is counterintuitive if one assumes that a critical size is necessary to trigger metamorphosis into the adult form, given that lower overall growth rates would delay metamorphosis and may potentially have a negative impact in fitness (Day and Rowe 2002). Taken together, developmental differences observed between groups indicate that increased pressures on larval survival in ephemeral ponds may have resulted in accelerated developmental rates at the expense of growth, which is adaptive if the benefits of developing faster and evolving lower threshold sizes are higher than the costs they impinge on growth rates. Importantly, these differences were detected under constant water-level conditions, suggesting that evolutionary responses to increased desiccation risks partly involve an evolutionary shift in mean values toward more accelerated development.

At the level of developmental plasticity, comparisons between species breeding in ephemeral and permanent ponds showed that both groups showed a similar reduction in developmental period in response to drying conditions. However, the nature of plastic responses actually depended on the breeding habitat: whereas species breeding in ephemeral ponds showed pronounced reductions in size at metamorphosis in response to drying conditions, this was generally not observed in species inhabiting permanent ponds (Fig. 4b). Even though a reduction in mean growth rate may be partly explained by increased stressful conditions when ponds are drying, this should not result in significant differences between species breeding in ephemeral and permanent ponds. Instead, contrasting patterns between groups suggest that species breeding in ephemeral ponds allocate resources preferentially to development rather than growth (Figs. 1c and 4c; Wilbur and Collins 1973; De Witt et al. 1998; Harris 1999).

### Evolutionary trade-offs and constraints

Development and growth involve two very distinct physiological processes, where the former is essentially associated with cell differentiation while the second is primarily determined by cell proliferation and growth (Smith-Gill and Berven 1979). Although these processes are intrinsically connected, some degree of independence between them must ultimately account for the diversity of developmental strategies observed across anuran species (Fig. 3), as predicted by Wilbur and Collin's (1973) model. Our analyses are particularly relevant in this context because species under strong selection for faster development during larval stages apparently maximize their developmental rates at the expense of growth, which suggests a trade-off between development and growth (Fig. 1c). Several lines of evidence support this conclusion. For instance, these species exhibit significantly slower mean growth rates in analyses controlling for differences in developmental rates under constant water-level conditions. In addition, the association between developmental rates and mean growth rates is nearly flat in this group, contrasting with the positive association between these variables observed in species breeding in permanent ponds, as indicated by the nearly significant interaction in the general linear model.

Furthermore, contrasting plastic responses to drying conditions indicate that species breeding in ephemeral ponds cannot increase mean growth rates under stressful conditions, which is expected if mean growth rates under constant water-level conditions are close to a physiological limit. The maximum observed increase in mean growth rates due to plasticity was 6.9% in species from ephemeral ponds and 74.5% in species breeding in permanent ponds. The mean response within the sample that showed an increase in mean growth rates (*n* = 16 cases) provided a similar picture, an average (±SE) increase of 3.6 ± 0.6% in species from ephemeral ponds versus a 29.1 ± 8.3% increase in their counterparts from permanent ponds (*t*_14_ = 3.5, *P* = 0.0036). This provides compelling evidence that anuran species breeding in ephemeral ponds are maximizing mean growth rates and consequently show little plasticity in this trait (Newman 1988; Reques and Tejedo 1997). Conversely, long-lasting pond breeders can increase mean growth rates under desiccation risks, hence mean growth rates under constant water-level conditions are apparently not being maximized (see also Wilbur 1987; Semlitsch and Wilbur 1988; Boone et al. 2004). Interestingly, some clades with facultative or obligate carnivory within the families Scaphiopodidae (Pfenning 1992) and Ceratophryidae (Cei 1980) breed in ephemeral ponds and have very short larval periods (Buchholz and Hayes 2002; Marangoni et al. 2009), suggesting that these diet shifts might have resulted from selection on increased mean growth rates.

While most species breeding in ephemeral ponds showed similar responses to drying conditions (a reduction in both mass at metamorphosis and mean growth rate), considerable variation in the nature and the magnitude of plastic responses was observed across species breeding in permanent ponds. Many species did not increase their developmental rates in response to drying conditions, even though some of these species exhibited a reduction in body size at metamorphosis of almost 40% (Fig. 4b). Conversely, other species showed important reductions in developmental time and were also able to increase mean growth rates as a compensatory response (Fig. 4c), suggesting that the primary target of selection in these species is body mass at metamorphosis. Importantly, this response was observed in distantly related taxa (Fig. S3), which explains why conventional statistics assuming a star phylogeny, provided a better fit in analyses of mean growth rates. Patterns observed in this subset agree with observations carried out in butterflies and damselflies, which exhibit a shortening in developmental period while simultaneously reducing size and accelerating mean growth rates (Nylin et al. 1996; Strobbe and Stoks 2004; De Block et al. 2008). In summary, our results show that plastic responses are constrained in species with accelerated developmental rates and suggest that they are indeed developing near their maximum physiological capacities. A similar comparative approach may shed light on the generality of this observation across other biological systems in which developmental rates are expected to be under selection.

### Phylogenetic effects

Absolute limits to performance certainly exist, but this does not account for the interspecific differences observed in developmental strategies. Although differences may be partly due to the stressful physical and biotic conditions of desiccating ponds, our results suggest that an important fraction of the variation can be attributed to phylogenetic history. For example, bufonids in general metamorphose at very small body sizes, whereas the opposite pattern is true for scaphiopodids (Fig. 2, see also Werner 1986). It is possible that contrasting larval sizes reflect differences in fecundity across species, hence more detailed information on maternal investment, egg, and clutch size may shed light on the relevant mechanisms underlying developmental differences between species (Wells 2007, pp. 494-515). In addition, physiological constraints may differ across taxa (e.g., rudimentary nonfunctional lungs are characteristic of bufonid larvae; Ultsch et al. 1999 and references therein).

Understanding which factors ultimately explain these differences is crucial to determine if and how anuran species may respond to increasing risks of desiccation. Even though comparative studies are strictly correlational and may provide limited information on the mechanisms underlying species developmental differences, phylogenetic information can be valuable for predictive purposes, because closely related taxa seem to employ similar developmental strategies and may also potentially share the same physiological limitations.

## Supporting Information

**Table S1.** Dataset

**Table S2.** Regression models.

**Figure S1.** Growth trajectory of two hypothetical species and definition of mean growth rate.

**Figure S2.** Developmental plasticity estimates.

**Figure S3.** Phylogenetic hypothesis and phenotypic plasticity.

## References

[b1] Abrams PA, Leimar O, Nylin S, Wiklund C (1996). The effect of flexible growth rates on optimal sizes and development times in a seasonal environment. Am. Nat..

[b2] Adams MJ (2000). Pond permanence and the effects of exotic vertebrates on anurans. Ecological Applications.

[b3] Arendt JD (1997). Adaptive intrinsic growth rates: an integration across taxa. Quat. Rev. Biol..

[b4] Babbitt KJ, Baber MJ, Tarr TL (2003). Patterns of larval amphibian distribution along a wetland hydroperiod gradient. Can. J. Zool..

[b5] Blomberg SP, Garland T, Ives AR (2003). Testing for phylogenetic signal in comparative data: behavioral traits are more labile. Evolution.

[b6] Boone MD, Little EE, Semlitsch RD (2004). Overwintered bullfrog tadpoles negatively affect salamanders and anurans in native amphibian communities. Copeia.

[b7] Brady LD, Griffiths RA (2000). Developmental responses to pond desiccation in tadpoles of the British anuran amphibians (*Bufo bufo*, *B. calamita* and *Rana temporaria*). J. Zool..

[b8] Buchholz DR, Hayes TB (2002). Evolutionary patterns of diversity in spadefoot toad metamorphosis (Anura: Pelobatidae). Copeia.

[b9] Burnham KP, Anderson DR (2002). Model selection and multi-model inference: a practical information-theoretic approach.

[b10] Cei JM (1980). Amphibians of Argentina. Monitore Zoologico Italiana (N.S.). Monografia.

[b11] Day T, Rowe L (2002). Developmental thresholds and the evolution of reaction norms for age and size at life-history transitions. Am. Nat..

[b12] De Block M, McPeek MA, Stoks R (2008). Life-history evolution when *Lestes* damselflies invaded vernal ponds. Evolution.

[b13] Denver RJ (1997). Proximate mechanisms of phenotypic plasticity in amphibian metamorphosis. Am. Zool..

[b14] De Witt TJ, Sih A, Wilson DS (1998). Costs and limits of phenotypic plasticity. Trends Ecol. Evol..

[b15] Doughty P, Reznick DN, DeWitt T, Scheiner SM (2004). Patterns and analysis of adaptive phenotypic plasticity in animals. Phenotypic plasticity: functional and conceptual approaches.

[b16] Faivovich J, Haddad CFB, García PCA, Frost DR, Campbell JA, Wheeler WC (2005). Systematic review of the frog family Hylidae, with special reference to Hylinae: phylogenetic analysis and taxonomic revision. Bull. Am. Mus. Nat. Hist..

[b17] Frost DR, Grant T, Faivovich J, Bain RH, Haas A, Haddad CFB, de Sá RO, Channing A, Wilkinson M, Donnellan SC (2006). The amphibian tree of life. Bull. Am. Mus. Nat. Hist..

[b18] García-París M, Buchholz DR, Parra-Olea G (2003). Phylogenetic relationships of Pelobatoidea re-examined using mtDNA. Mol. Phylogenet. Evol..

[b19] Garland T, Bennett AF, Rezende EL (2005). Phylogenetic approaches in comparative physiology. J. Exp. Biol..

[b20] Garland T, Díaz-Uriarte R (1999). Polytomies and independent contrasts: an examination of the bounded degrees of freedom approach. Systematic Biology.

[b21] Gomes FR, Rezende EL, Grizante MB, Navas CA (2009). The evolution of jumping performance in anurans: morphological correlates and ecological implications. J. Evol. Biol..

[b22] Gotthard K, Nylin S (1995). Adaptive plasticity and plasticity as an adaptation: a selective review of plasticity in animal morphology and life history. Oikos.

[b23] Harris RN, McDiarmid RW, Altig R (1999). The anuran tadpole: evolution and maintenance. Tadpoles: the biology of anuran larvae.

[b24] Hillis DM, Wilcox TP (2005). Phylogeny of the New World true frogs (*Rana*). Mol. Phylogenet. Evol..

[b25] Johansson F, Stoks R, Rowe L, De Block M (2001). Life history plasticity in a damselfly: effects of combined time and biotic constraints. Ecology.

[b26] Lannoo M (2005). Amphibian declines: the conservation status of United States species.

[b27] Leips J, McManus MG, Travis J (2000). Response of treefrog larvae to drying ponds: comparing temporary and permanent pond breeders. Ecology.

[b28] Lind MI, Johansson F (2007). The degree of adaptive phenotypic plasticity is correlated with the spatial environmental heterogeneity experienced by island populations of *Rana temporaria*. J. Evol. Biol..

[b29] Marangoni F, Schaefer E, Cajade R, Tejedo M (2009). Growth marks formation and chronology of two neotropical anuran species. J. Herpetol..

[b30] Merilä J, Laurila A, Lindgren B (2004). Variation in the degree and costs of adaptive phenotypic plasticity among *Rana temporaria* populations. J. Evol. Biol..

[b31] Morey S, Reznick DN (2004). The relationship between habitat permanence and larval development in California spadefoot toads: field and laboratory comparisons of developmental plasticity. Oikos.

[b32] Newman RA (1988). Adaptive plasticity in development of *Scaphiopus couchii* tadpoles in desert ponds. Evolution.

[b33] Newman RA (1992). Adaptive plasticity in amphibian metamorphosis. Bioscience.

[b34] Nunney L (1996). The response to selection for fast larval development in *Drosophila melanogaster* and its effect on adult weight: an example of a fitness trade-off. Evolution.

[b35] Nylin S, Gotthard K, Wiklund C (1996). Reaction norms and size at maturity in *Lasiommata* butterflies: predictions and tests. Evolution.

[b36] Nylin S, Gotthard K (1998). Plasticity in life-history traits. Annu. Rev. Entomol..

[b37] Pagel M (1992). A method for the analysis of comparative data. J. Theor. Biol..

[b38] Pauly GB, Hillis DM, Cannatella DC (2004). The history of a nearctic colonization: molecular phylogenetics and biogeography of the nearctic toads (*Bufo*). Evolution.

[b39] Pfenning DW (1992). Proximate and functional causes of polyphenism in an anuran tadpole. Funct. Ecol..

[b40] Purvis A, Garland T (1993). Polytomies in comparative analyses of continuous data. Systematic Biology.

[b41] Read K, Scott Keogh J, Scott IAW, Dale Roberts J, Doughty P (2001). Molecular phylogeny of the Australian frog genera *Crinia, Geocrinia*, and allied taxa (Anura: Myobatrachidae). Mol. Phylogenet. Evol..

[b42] Reques R, Tejedo M (1997). Reaction norms for metamorphic traits in natterjack toads to larval density and pond duration. J. Evol. Biol..

[b43] Richardson JML (2001). The relative roles of adaptation and phylogeny in determination of larval traits in diversifying anuran lineages. Am. Nat..

[b44] Richter-Boix A, Llorente GA, Montori A (2006). A comparative analysis of the adaptive developmental hypothesis in six Mediterranean anuran species along a pond permanency gradient. Evol. Ecol. Res..

[b45] Roelants K, Gower DJ, Wilkinson M, Loader SP, Biju SD, Guillaume K, Linde M, Bossuyt F (2007). Global patterns of diversification in the history of modern amphibians. Proc. Natl. Acad. Sci. U. S. A..

[b46] Rosenberg MS, Adams DC, Gurevitch J (2000). MetaWin: statistical software for meta-analysis.

[b47] Schäuble CS, Moritz C, Slade RW (2000). A molecular phylogeny for the frog genus *Limnodynastes* (Anura: Myobatrachidae). Mol. Phylogenet. Evol..

[b48] Scott E (2005). A phylogeny of ranid frogs (Anura: Ranoidea: Ranidae) based on a simultaneous analysis of morphological and molecular data. Cladistics.

[b49] Semlitsch RD, Wilbur HM (1988). Effects of pond drying time on metamorphosis and survival in the salamander *Ambystoma talpoideum*. Copeia.

[b50] Smith-Gill SJ, Berven KA (1979). Predicting amphibian metamorphosis. Am. Nat..

[b51] Stearns SC, Koella JC (1986). The evolution of phenotypic plasticity in life-history traits: predictions of reaction norms for age and size at maturity. Evolution.

[b52] Stoks R, De Block M, McPeek MA (2006). Physiological costs of compensatory growth in a damselfly. Ecology.

[b53] Strobbe F, Stoks R (2004). Life history reaction norms to time constraints in a damselfly: differential effects on size and mass. Biol. J. Linn. Soc. Lond. B.

[b54] Turkheimer FE, Hinz R, Cunningham VJ (2003). On the undecidability among kinetic models: from model selection to model averaging. J. Cereb. Blood Flow Metab..

[b55] Ultsch GR, Bradford DF, Freda J, McDiarmid RW, Altig R (1999). Physiology: coping with the environment. Tadpoles: the biology of anuran larvae.

[b56] Veith M, Kosuch J, Vences M (2003). Climatic oscillations triggered post-Messinian speciation of Western Paleartic brown frogs (Amphibia, Anura, Ranidae). Phylogenetics and Evolution.

[b57] Wells KD (2007). The ecology and behavior of amphibians.

[b58] Werner EE (1986). Amphibian metamorphosis: growth rate, predation risk, and the optimal size at transformation. Am. Nat..

[b59] Wiens JJ, Graham CH, Moen DS, Smith SA, Reeder TW (2006). Evolutionary and ecological causes of the latitudinal diversity gradient in Hylid frogs: treefrog trees unearth the roots of high tropical diversity. Am. Nat..

[b60] Wiens JJ (2007). Global patterns of diversification and species richness in amphibians. Am. Nat..

[b61] Wilbur HM (1987). Regulation of structure in complex systems: experimental temporary pond communities. Ecology.

[b62] Wilbur HM, Collins JP (1973). Ecological aspects of amphibian metamorphosis. Science.

[b63] Woodward BD (1983). Predator-prey interactions and breeding-pond use of temporary-pond species in a desert anuran community. Ecology.

