# Supplementary material for: Evolution and plasticity of anuran larval development in response to desiccation. A comparative analysis
Source: Ecol Evol. 2011 Sep;1(1):15–25. doi: 10.1002/ece3.2 (PMC3287374; doi:10.1002/ece3.2)
Supplement: Supplementary file 1 [file ece30001-0015-SD1.doc]

**Supporting Information**

**EVOLUTION AND PLASTICITY OF ANURAN LARVAL development IN RESPONSE TO DESICCATION. a comparative analysis**

**Alex Richter-Boix, Miguel Tejedo and Enrico L. Rezende**

MATERIAL INCLUDED:

Table S1. Dataset

Table S2. Regression Models. Results section *Phenotypic Plasticity*

Figure S1. Growth trajectory of two hypothetical species and definition of mean growth rate.

Figure S2. Developmental plasticity estimates

Figure S3. Phylogenetic hypothesis and phenotypic plasticity

References

Table S1. Dataset

| Species | Code | Risk | *n* | | Developmental time (days)^§^ | | Body mass (g) ^§^,*** | | Mean Growth rates (g/day * 1000) ^§^ | | Time plasticity | | Mass plasticity | | Growth plasticity | | Ref |
| --- | --- | --- | --- | --- | --- | --- | --- | --- | --- | --- | --- | --- | --- | --- | --- | --- | --- |
|  |  |  | cte | dry | cte | dry | cte | dry | cte | dry | % | effect | % | effect | % | effect |  |
| *Alytes obstetricans* | Ao | Low | 18 | 14 | 135.17 ± 16.45 | 109.36 ± 10.79 | 1.269 ± 0.153 | 0.995 ± 0.111 | 9.386 ± 1.607 | 9.101 ± 1.355 | -19.1 | -1.76 | -21.6 | -1.96 | -3.0 | -0.18 | 1 |
| *Scaphiopus couchii* | Sc | High | 3 | 2 | 7.17 ± 0.29 | 7.00 ± 0.00 | 0.454 ± 0.192 | 0.423 ± 0.304 | 63.335 ± 26.981 | 60.485 ± 43.415 | -2.3 | -0.51 | -6.7 | -0.09 | -4.5 | -0.06 | 2 |
| *Spea hammondii* | S7 | High | 3 | 3 | 71.33 ± 6.56 | 44.00 ± 16.37 | 6.137 ± 3.531 | 2.308 ± 0.825 | 86.034 ± 50.151 | 52.447 ± 27.064 | -38.3 | -1.75 | -62.4 | -1.19 | -39.0 | -0.67 | 2 |
| *Spea hammondii* | S6 | High | 3 | 3 | 61.42 ± 1.46 | 48.79 ± 2.55 | 4.743 ± 0.844 | 1.385 ± 0.178 | 77.231 ± 13.861 | 28.379 ± 3.933 | -20.6 | -4.86 | -70.8 | -4.41 | -63.3 | -3.84 | 3 |
| *Spea hammondii* | S5 | High | 8 | 8 | 54.60 ± 2.26 | 43.90 ± 1.13 | 2.800 ± 0.566 | 1.700 ± 0.283 | 51.282 ± 10.576 | 38.724 ± 6.520 | -19.6 | -5.66 | -39.3 | -2.33 | -24.5 | -1.35 | 3 |
| *Spea hammondii* | S4 | High | 3 | 3 | 44.84 ± 1.02 | 37.15 ± 0.47 | 2.702 ± 0.187 | 1.568 ± 0.109 | 60.276 ± 4.397 | 42.196 ± 2.989 | -17.1 | -7.74 | -42.0 | -5.92 | -30.0 | -3.85 | 3 |
| *Spea hammondii* | S3 | High | 3 | 3 | 40.00 ± 1.18 | 34.54 ± 1.38 | 3.767 ± 0.887 | 2.046 ± 0.203 | 94.175 ± 22.344 | 59.227 ± 6.323 | -13.6 | -3.40 | -45.7 | -2.14 | -37.1 | -1.70 | 3 |
| *Spea hammondii* | S2 | High | 3 | 3 | 40.30 ± 1.21 | 31.5 ± 2.25 | 3.850 ± 0.814 | 1.800 ± 0.208 | 95.533 ± 20.403 | 57.143 ± 7.760 | -21.8 | -3.89 | -53.2 | -2.76 | -40.2 | -1.99 | 3 |
| *Spea hammondii* | S1 | High | 3 | 3 | 61.15 ± 1.04 | 48.54 ± 2.29 | 4.780 ± 0.758 | 1.360 ± 0.162 | 78.163 ± 12.463 | 28.019 ± 3.598 | -20.6 | -5.32 | -71.5 | -5.23 | -64.2 | -4.37 | 3 |
| *Pelobates cultripes* | Pc | High | 3 | 3 | 172.34 ± 6.25 | 165.95 ± 4.83 | 2.399 ± 0.175 | 1.658 ± 0.243 | 13.920 ± 1.132 | 9.990 ± 1.492 | -3.7 | -0.92 | -30.9 | -2.80 | -28.2 | -2.37 | 4 |
| *Pelodytes punctatus* | Pp | High | 38 | 26 | 73.89 ± 20.73 | 62.00 ± 12.12 | 0.212 ± 0.064 | 0.159 ± 0.041 | 2.865 ± 1.184 | 2.563 ± 0.832 | -16.1 | -0.66 | -24.9 | -0.93 | -10.5 | -0.28 | 1 |
| *Pelodytes ibericus* | Pi | High | 3 | 3 | 105.57 ± 1.65 | 101.41 ± 0.95 | 0.423 ± 0.032 | 0.306 ± 0.052 | 4.012 ± 0.311 | 3.019± 0.517 | -3.9 | -2.47 | -27.7 | -2.16 | -24.7 | -1.86 | 5 |
| *Limnodynastes tasmaniensis* | Lt | Low | 6 | 6 | 122.00 ± 14.70 | 122.00 ± 19.60 | 0.660 ± 0.098 | 0.460 ± 0.049 | 5.410 ± 1.034 | 3.770 ± 0.727 | 0.0 | 0.00 | -30.3 | -2.38 | -30.3 | -1.69 | 6 |
| *Pseudophryne australis* | Pa | High | 18 | 28 | 63.59 ± 15.65 | 66.93 ± 12.18 | 0.081 ± 0.018 | 0.091 ± 0.014 | 1.272 ± 0.419 | 1.359 ± 0.321 | 5.3 | 0.24 | 12.5 | 0.65 | 6.9 | 0.24 | 7 |
| *Crinia signifera* | Cs | High | 6 | 6 | 31.00 ± 1.22 | 28.00 ± 3.67 | 0.100 ± 0.012 | 0.075 ± 0.012 | 3.226 ± 0.415 | 2.678 ± 0.568 | -9.7 | -1.01 | -25.0 | -1.87 | -17.0 | -1.02 | 6 |
| *Bufo calamita* | 1B | High | 3 | 3 | 48.00 ± 5.19 | 47.00 ± 2.60 | 0.068 ± 0.019 | 0.067 ± 0.007 | 1.417 ± 0.425 | 1.425 ± 0.167 | -2.1 | -0.19 | -1.5 | -0.06 | 0.6 | 0.02 | 8 |
| *Bufo calamita* | 2B | High | 3 | 3 | 57.00 ± 2.08 | 55.00 ± 3.46 | 0.090 ± 0.017 | 0.063 ± 0.010 | 1.579 ± 0.309 | 1.145 ± 0.202 | -3.5 | -0.56 | -30.0 | -1.51 | -27.5 | -1.33 | 8 |
| *Bufo calamita* | 3B | High | 16 | 16 | 61.18 ± 9.81 | 56.12 ± 6.53 | 0.110 ± 0.026 | 0.106 ± 0.020 | 1.797 ± 0.516 | 1.897 ± 0.420 | -8.3 | -0.59 | -3.2 | -0.14 | 5.6 | 0.21 | 1 |
| *Bufo calamita* | 4B | High | 5 | 5 | 58.05 ± 1.08 | 58.63 ± 1.08 | 0.122 ± 0.052 | 0.121 ± 0.004 | 2.107 ± 0.098 | 2.066 ± 0.075 | 1.0 | 0.48 | -0.9 | -0.22 | -1.9 | -0.42 | 5 |
| *Bufo calamita* | 5B | High | 3 | 3 | 32.25 ± 0.60 | 30.55 ± 0.15 | 0.078 ± 0.004 | 0.076 ± 0.001 | 2.412 ± 0.123 | 2.489 ± 0.051 | -5.2 | -3.11 | -2.2 | -0.48 | 3.2 | 0.65 | 9 |
| *Bufo calamita* | 6B | High | 3 | 3 | 30.20 ± 0.44 | 29.46 ± 0.24 | 0.114 ± 0.016 | 0.117 ± 0.004 | 3.788 ± 0.546 | 3.975 ± 0.127 | -2.4 | -1.66 | 2.4 | 0.18 | 4.9 | 0.38 | 9 |
| *Bufo calamita* | 7B | High | 11 | 12 | 29.53 ± 1.64 | 29.10 ± 2.80 | 0.108 ± 0.010 | 0.110 ± 0.013 | 3.652 ± 0.388 | 3.775 ± 0.583 | -1.5 | -0.18 | 1.9 | 0.16 | 3.4 | 0.24 | 10 |
| *Bufo calamita* | 8B | High | 3 | 3 | 45.98 ± 3.34 | 44.63 ± 5.77 | 0.065 ± 0.022 | 0.064 ± 0.012 | 1.412 ± 0.485 | 1.446 ± 0.331 | -2.9 | -0.23 | -0.5 | -0.01 | 2.4 | 0.07 | 11 |
| *Bufo bufo* | b4 | Low | 3 | 3 | 76.52 ± 1.30 | 75.00 ± 1.62 | 0.124 ± 0.009 | 0.119 ± 0.009 | 1.625 ± 0.125 | 1.593 ± 0.128 | -2.0 | -0.82 | -3.9 | -0.41 | -2.0 | -0.20 | 5 |
| *Bufo bufo* | b3 | Low | 17 | 16 | 92.18 ± 6.16 | 90.50 ± 6.87 | 0.120 ± 0.023 | 0.104 ± 0.020 | 1.307 ± 0.268 | 1.153 ± 0.240 | -1.8 | -0.25 | -13.4 | -0.72 | -11.8 | -0.59 | 1 |
| *Bufo bufo* | b2 | Low | 3 | 3 | 61.00 ± 0.52 | 62.00 ± 1.73 | 0.085 ± 0.005 | 0.065 ± 0.009 | 1.393 ± 0.086 | 1.048 ± 0.143 | 1.6 | 0.63 | -23.5 | -2.23 | -24.8 | -2.34 | 8 |
| *Bufo bufo* | b1 | Low | 3 | 3 | 42.00 ± 0.35 | 41.70 ± 0.17 | 0.135 ± 0.014 | 0.110 ± 0.017 | 3.214 ± 0.331 | 2.638 ± 0.415 | -0.7 | -0.88 | -18.5 | -1.27 | -17.9 | -1.23 | 8 |
| *Bufo maculatus* | 2b | High | 9 | 8 | 27.51 ± 1.49 | 24.64 ± 3.01 | 0.028 ± 0.003 | 0.026 ± 0.003 | 1.024 ± 0.139 | 1.044 ± 0.183 | -10.4 | -1.17 | -8.7 | -0.71 | 2.0 | 0.12 | 12 |
| *Bufo maculatus* | 1b | High | 8 | 10 | 19.60 ± 1.88 | 17.83 ± 1.65 | 0.071 ± 0.009 | 0.060 ± 0.098 | 3.609± 0.579 | 3.362 ± 0.632 | -9.1 | -0.96 | -15.3 | -1.08 | -6.8 | -0.38 | 12 |
| *Bufo americanus* | B3 | Low | 12 | 12 | 54.60 ± 1.94 | 53.60 ± 3.46 | 0.130 ± 0.020 | 0.100 ± 0.030 | 2.381 ± 0.378 | 1.866 ± 0.575 | -1.8 | -0.34 | -23.1 | -1.13 | -21.6 | -1.02 | 13 |
| *Bufo americanus* | B2 | Low | 11 | 12 | 55.60 ± 2.77 | 53.10 ± 3.65 | 0.097 ± 0.023 | 0.120 ± 0.285 | 1.745 ± 0.426 | 2.260 ± 0.559 | -4.5 | -0.74 | 23.7 | 0.85 | 29.5 | 0.99 | 13 |
| *Bufo americanus* | B1 | Low | 7 | 12 | 33.80 ± 1.06 | 33.70 ± 1.04 | 0.177 ± 0.024 | 0.164 ± 0.021 | 5.237 ± 0.723 | 4.866 ± 0.635 | -0.3 | -0.09 | -7.3 | -0.57 | -7.1 | -0.53 | 14 |
| *Litoria aurea* | La | Low | 6 | 6 | 42.50 ± 3.67 | 41.75 ± 4.29 | 2.300 ± 0.980 | 1.500 ± 0.245 | 54.118 ± 23.524 | 35.928 ± 6.930 | -1.8 | -0.17 | -34.8 | -1.03 | -33.6 | -0.97 | 15 |
| *Pseudacris regilla* | Pr | Low | 5 | 3 | 96.00 ± 35.78 | 108.00 ± 112.58 | 0.700± 0.112 | 0.500 ± 0.121 | 7.292 ± 2.956 | 4.630 ± 4.955 | 12.5 | 0.15 | -28.6 | -1.51 | -36.5 | -0.62 | 16 |
| *Pseudacris triseriata* | Pt | High | 6 | 6 | 39.60 ± 7.10 | 38.50 ± 4.90 | 0.141 ± 0.017 | 0.142 ± 0.012 | 3.561 ± 0.772 | 3.688 ± 0.567 | -2.8 | -0.17 | 0.7 | 0.06 | 3.6 | 0.17 | 17 |
| *Isthmohyla pseudopuma* | Hp | High | 6 | 6 | 25.70 ± 1.25 | 24.94 ± 1.24 | 0.226 ± 0.024 | 0.202 ± 0.018 | 8.805 ± 1.025 | 8.114 ± 0.842 | -3.0 | -0.59 | -10.6 | -1.03 | -7.8 | -0.68 | 18 |
| *Hyla versicolor* | Hv | Low | 2 | 2 | 50.50 ± 7.78 | 29.50 ± 3.53 | 0.765 ± 0.113 | 0.780 ± 0.099 | 15.148 ± 3.235 | 26.441 ± 4.615 | -41.6 | -1.99 | 2.0 | 0.08 | 74.5 | 1.62 | 19 |
| *Hyla meridionalis* | Hm | Low | 19 | 15 | 144.47 ± 19.15 | 102.8 ± 10.67 | 0.803 ± 0.069 | 0.688 ± 0.078 | 5.559 ± 0.870 | 6.690 ± 1.033 | -28.8 | -2.54 | -14.4 | -1.56 | 20.3 | 1.17 | 1 |
| *Hyla savignyi* | Hs | Low | 3 | 4 | 42.00 ± 4.67 | 35.78 ± 2.33 | 0.303 ± 0.047 | 0.212 ± 0.062 | 7.214 ± 1.372 | 5.925 ± 1.775 | -14.8 | -1.51 | -30.0 | -1.36 | -17.9 | -0.67 | 20 |
| *Hoplobatrachus occipitalis* | Ho | High | 3 | 5 | 55.35 ± 1.03 | 54.77 ± 2.32 | 0.964 ± 0.042 | 0.879 ± 0.089 | 17.412 ± 0.818 | 16.042 ± 1.758 | -1.0 | -0.25 | -8.8 | -0.97 | -7.9 | -0.79 | 12 |
| *Pelophylax perezi* | Rp | Low | 18 | 11 | 129.17 ± 15.56 | 107.09 ± 3.94 | 0.737 ± 0.078 | 0.708 ± 0.070 | 5.710 ± 0.917 | 6.610 ± 0.696 | -17.1 | -1.71 | -4.0 | -0.38 | 15.8 | 1.04 | 1 |
| *Pelophylax lessonae* | Rl | Low | 3 | 3 | 48.77 ± 1.40 | 53.50 ± 2.01 | 0.941 ± 0.062 | 0.620 ± 0.171 | 19.297 ± 1.386 | 11.591 ± 3.231 | 9.7 | 2.19 | -34.1 | -1.99 | -39.9 | -2.48 | 21 |
| *Pelophylax esculenta* | Re | Low | 3 | 3 | 51.30 ± 5.11 | 49.90 ± 4.35 | 0.817 ± 0.121 | 0.596 ± 0.110 | 15.928 ± 2.836 | 11.945 ± 2.444 | -2.7 | -0.24 | -27.1 | -1.53 | -25.0 | -1.20 | 21 |
| *Rana temporaria* | r9 | Low | 5 | 5 | 34.80 ± 2.68 | 32.00 ± 1.34 | 0.600 ± 0.078 | 0.360 ± 0.045 | 17.241 ± 2.612 | 11.250 ± 1.475 | -8.0 | -1.19 | -40.0 | -3.40 | -34.7 | -2.55 | 22 |
| *Rana temporaria* | r8 | Low | 4 | 4 | 32.62 ± 2.85 | 30.57 ± 1.35 | 0.181 ± 0.020 | 0.180 ± 0.028 | 5.550 ± 0.772 | 5.888 ± 0.962 | -6.3 | -0.80 | -0.6 | -0.04 | 6.1 | 0.34 | 23 |
| *Rana temporaria* | r7 | Low | 3 | 3 | 62.00 ± 1.39 | 59.00 ± 0.87 | 0.175 ± 0.017 | 0.135 ± 0.012 | 2.822 ± 0.286 | 2.288 ± 0.208 | -4.8 | -2.08 | -22.9 | -2.14 | -18.9 | -1.71 | 8 |
| *Rana temporaria* | r6 | Low | 3 | 3 | 37.50 ± 0.87 | 37.00 ± 0.35 | 0.230 ± 0.026 | 0.220 ± 0.026 | 6.133 ± 0.707 | 5.946 ± 0.704 | -1.3 | -0.61 | -4.3 | -0.31 | -3.1 | -0.21 | 8 |
| *Rana temporaria* | r5 | Low | 10 | 10 | 24.38 ± 0.59 | 23.68 ± 0.44 | 0.386 ± 0.010 | 0.348 ± 0.011 | 15.821 ± 0.566 | 14.716 ± 0.545 | -2.8 | -1.27 | -9.6 | -3.33 | -7.0 | -1.90 | 24 |
| *Rana temporaria* | r4 | Low | 10 | 10 | 22.50 ± 0.60 | 22.52 ± 0.606 | 0.365 ± 0.014 | 0.346 ± 0.009 | 16.225 ± 0.763 | 15.386 ± 0.562 | 0.1 | 0.03 | -5.1 | -1.53 | -5.2 | -1.20 | 24 |
| *Rana temporaria* | r3 | Low | 8 | 8 | 31.10 ± 1.41 | 31.90 ± 1.13 | 0.560 ± 0.057 | 0.440 ± 0.042 | 18.006 ± 1.995 | 13.793 ± 1.417 | 2.6 | 0.59 | -21.4 | -2.27 | -23.4 | -2.30 | 25 |
| *Rana temporaria* | r2 | Low | 8 | 8 | 31.00 ± 2.26 | 31.75 ± 1.41 | 0.570 ± 0.071 | 0.430 ± 0.848 | 18.387 ± 2.646 | 13.543 ± 2.740 | 2.4 | 0.38 | -24.6 | -1.70 | -26.3 | -1.70 | 25 |
| *Rana temporaria* | r1 | Low | 8 | 8 | 31.60 ± 1.84 | 31.85 ± 1.98 | 0.460 ± 0.071 | 0.395 ± 0.099 | 14.557 ± 2.393 | 12.402 ± 3.202 | 0.8 | 0.12 | -14.1 | -0.71 | -14.8 | -0.72 | 25 |
| *Rana aurora* | Ra | Low | 5 | 3 | 114.00 ± 125.22 | 117 ± 171.47 | 4.000 ± 1.677 | 2.400 ± 3.308 | 35.088 ± 41.253 | 20.513 ± 41.271 | 2.6 | 0.02 | -40.0 | -0.59 | -41.5 | -0.31 | 16 |
| *Rana sylvatica* | Rs | High | 6 | 6 | 72.00 ± 4.90 | 76.00 ± 4.90 | 0.585 ± 0.115 | 0.585 ± 0.135 | 8.125 ± 1.692 | 7.697 ± 1.841 | 5.6 | 0.75 | 0.0 | 0.00 | -5.3 | -0.22 | 26 |
| *Rana blairi* | Rb | Low | 3 | 3 | 48.76 ± 3.96 | 44.71 ± 3.31 | 1.612 ± 0.400 | 0.965 ± 0.334 | 33.072 ± 8.640 | 21.576 ± 7.652 | -8.3 | -0.89 | -40.2 | -1.40 | -34.8 | -1.13 | 27 |
| *R. blairi × sphenocephala F2* | F2 | Low | 9 | 9 | 93.30 ± 13.05 | 113.00 ± 26.10 | 0.606 ± 0.129 | 0.618 ± 0.258 | 6.498 ± 1.655 | 5.469 ± 2.612 | 21.1 | 0.91 | 1.9 | 0.05 | -15.8 | -0.45 | 27 |
| *R. sphenocephala × blairi* | h2 | Low | 10 | 10 | 81.70 ± 11.23 | 91.00 ± 11.23 | 0.588 ± 0.111 | 0.425 ± 0.111 | 7.195 ± 1.680 | 4.672 ± 1.349 | 11.4 | 0.79 | -27.7 | -1.40 | -35.1 | -1.59 | 27 |
| *R. blairi × sphenocephala* | h1 | Low | 10 | 10 | 86.00 ± 12.30 | 84.30 ± 15.91 | 0.583 ± 0.122 | 0.515 ± 0.157 | 6.784 ± 1.716 | 6.105 ± 2.192 | -2.0 | -0.11 | -11.8 | -0.47 | -10.0 | -0.33 | 27 |
| *Rana sphenocephala* | R4 | Low | 10 | 10 | 87.20 ± 12.30 | 70.70 ± 15.91 | 0.561 ± 0.122 | 0.561 ± 0.157 | 6.429 ± 1.665 | 7.929 ± 2.850 | -18.9 | -1.11 | 0.0 | 0.00 | 23.3 | 0.62 | 27 |
| *Rana sphenocephala* | R3 | Low | 3 | 3 | 46.84 ± 3.12 | 45.06 ± 4.80 | 1.707 ± 0.316 | 1.244 ± 0.490 | 36.434 ± 7.171 | 27.620 ± 11.265 | -3.8 | -0.35 | -27.1 | -0.90 | -24.2 | -0.75 | 27 |
| *Rana sphenocephala* | R2 | Low | 4 | 4 | 75.00 ± 6.00 | 60.00 ± 1.00 | 0.330 ± 0.036 | 0.355 ± 0.040 | 4.400 ± 0.595 | 5.917 ± 0.674 | -20.0 | -3.03 | 7.6 | 0.57 | 34.5 | 2.07 | 28 |
| *Rana sphenocephala* | R1 | Low | 8 | 10 | 53.70 ± 2.83 | 46.10 ± 3.16 | 2.055 ± 0.492 | 0.730 ± 0.582 | 38.268 ± 9.384 | 15.835 ± 12.668 | -14.2 | -2.40 | -64.5 | -2.32 | -58.6 | -1.88 | 14 |

*Code:* refers to the phylogeny depicted in the left of Fig. A3. *Risk*: Desiccation risk. *High*: species exposed to a high risk of larval mortality by pond desiccation, including ephemeral and temporary ponds that hold water for only a few weeks or months and dry each year, occasionally with several dryings and refills per season. *Low:* Species exposed to a lower risk of pond desiccation, basically permanent ponds holding water year-round in most years with rare events of desiccation. *n*: sample size. *Cte* and *dry*. Correspond to drying and constant conditions. *Trait Plasticity*. These estimates were calculated as *%* percentage: $100\times\frac{(trait_{d}-trait_{c})}{trait}_{c}$where *d* and *c* correspond to drying (*dry*) and constant (*cte*) conditions, respectively. *Effect*: means Hedges’ *d* estimate of effect sizes, obtained with MetaWin 2.1 (Rosenberg et al. 2000). *Ref*: References: 1. Richter-Boix et al. (2006), 2. Morey and Reznick (2004), 3. Denver et al. (1998), 4. Tejedo, M., Graciá, E., and Iriarte, C. (unpublished), 5. Reques, R. and Tejedo, M. (unpublished), 6. Lane and Mahony (2002), 7. Thumm and Mahony (2006), 8. Brady and Griffiths (2000), 9. Tejedo and Reques (1994), 10. Reques and Tejedo (1997), 11. Tejedo, M., Iriarte, C and Graciá, E. (unpublished), 12. Spieler (2000), 13. Pearman (1993), 14. Boone, M.D., Little E.E., and Semlitsch R.D., unpublished data, 15. Hamer et al. (2002), 16. Adams (2000), 17. Durnin and Smith (2001), 18. Crump (1988), 19. Kiesecker and Skelly (2001), 20. Blaustein et al. (1999), 21. Semlitsch and Reyer (1992), 22. Laurila and Kujasalo (1999), 23. Loman (1999), 24. Laurila et al. (2002), 25. Pakkasma and Laurila (2004), 26. Rowe and Dunson (1995), 27. Parris (2000), 28. Ryan and Winne (2001).

^§^ Values represent mean ± SD (*Note*: SD for mean growth rates was calculated employing the approximation to estimate variances from a ratio


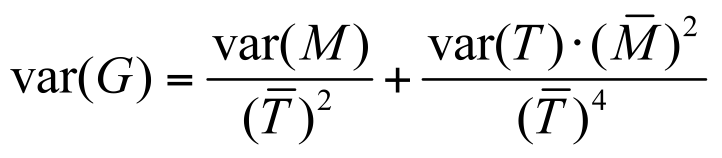


, where the covariance between *M* and *T* was assumed to be negligible; Kendall and Stuart 1977).

**Hyla regilla* and *Rana aurora* values on mass at metamorphosis and growth rates were expressed as ml and ml day^-1^, respectively.

Table S2. Results section *Phenotypic Plasticity*: Developmental plasticity due to desiccation differences in developmental rates, body mass at metamorphosis and mean growth rates of species from *High risk* versus *Low risk* of desiccation. Note that 25 degrees of freedom in phylogenetic analyses have been subtracted to account for the soft polytomies in our phylogenetic hypothesis. The models with the best fit are highlighted in bold.

| Plasticity | Model | *Intercept* ^§^ | *Desiccation risk* | *AIC* | *AIC_w_* |
| --- | --- | --- | --- | --- | --- |
| Developmental rates | Conventional | *t*_60_ = 3.63, *P* = 0.0006 | *t*_60_ = – 0.98, *P* = 0.322 | 510.01 | 0.00 |
|  | **Phylogenetic** | ***t*_35_ = 0.62, *P* = 0.539** | ***t*_35_ = – 0.18, *P* = 0.858** | **491.04** | **1.00** |
| Mean growth rates | **Conventional** | ***t*_60_ = – 3.30, *P* = 0.0016** | ***t*_60_ = 0.51, *P* = 0.614** | **563.29** | **0.999** |
|  | Phylogenetic | *t*_35_ = – 0.35, *P* = 0.728 | *t*_35_ = – 0.93, *P* = 0.359 | 569.04 | 0.001 |
| Mass at metamorphosis | Conventional | *t*_60_ = – 5.33, *P* < 0.0001 | *t*_60_ = 0.54, *P* = 0.592 | 544.73 | 0.05 |
|  | **Phylogenetic** | ***t*_35_ = – 0.70, *P* = 0.489** | ***t*_35_ = – 1.40, *P* = 0.170** | **542.10** | **0.95** |

^§^ Tests the hypothesis that the overall plastic response to desiccation is significantly different than zero.

Figure S1. Growth trajectory of two hypothetical species and definition of mean growth rate.


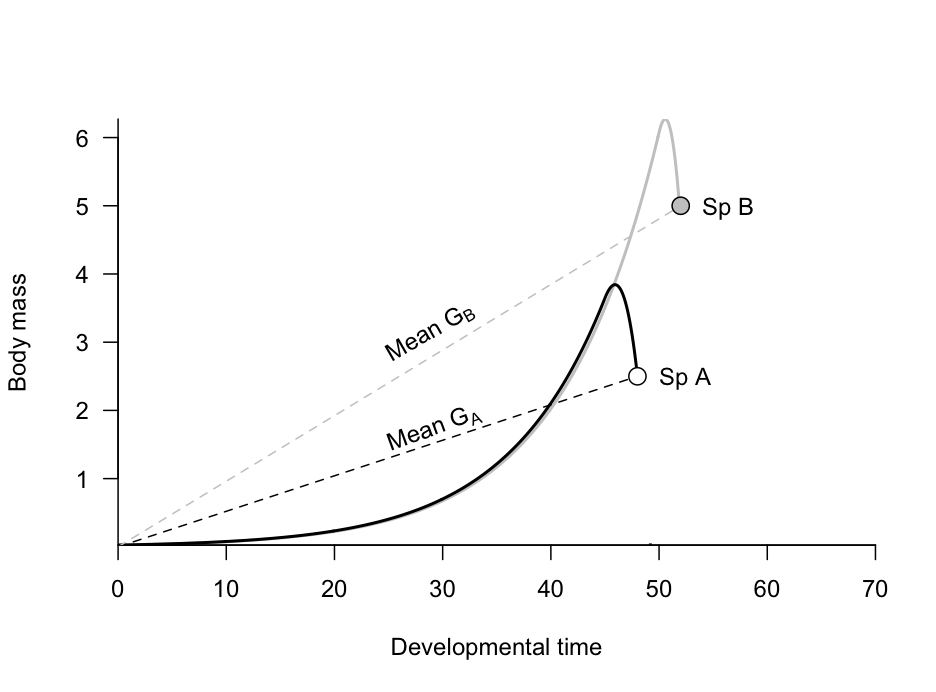
The non-linear nature of tadpole growth (continuous lines) requires incorporating three basic parameters: initial size (W_0_), size-specific growth rate (g=dW/dt) and an exponential decay of the size-specific growth rate, (-α) (see Harris 1999). Variations in any of these parameters may affect the final size at metamorphosis. The metric we calculate as *body mass at metamorphosis* */ developmental period* estimates the average rate of growth throughout the entire developmental period (dotted lines). As such, this metric provides an estimate of how fast or how slow different species grow on average, but it does not provide any information regarding the true growth trajectory of these species.

Figure S2. Developmental plasticity estimates

Plasticity calculated as percentages (see *Methods*) and Hedges’ *d* estimate of effect sizes, obtained with MetaWin 2.1 (Rosenberg et al. 2000). Both estimates are highly correlated for developmental time, body mass at metamorphosis and mean growth rates, as shown by the regression lines (linear regression through the origin, *P* < 0.0001 in all cases). Conclusions in the main text remain unchanged when effect sizes were employed as estimates of plasticity (results not shown).


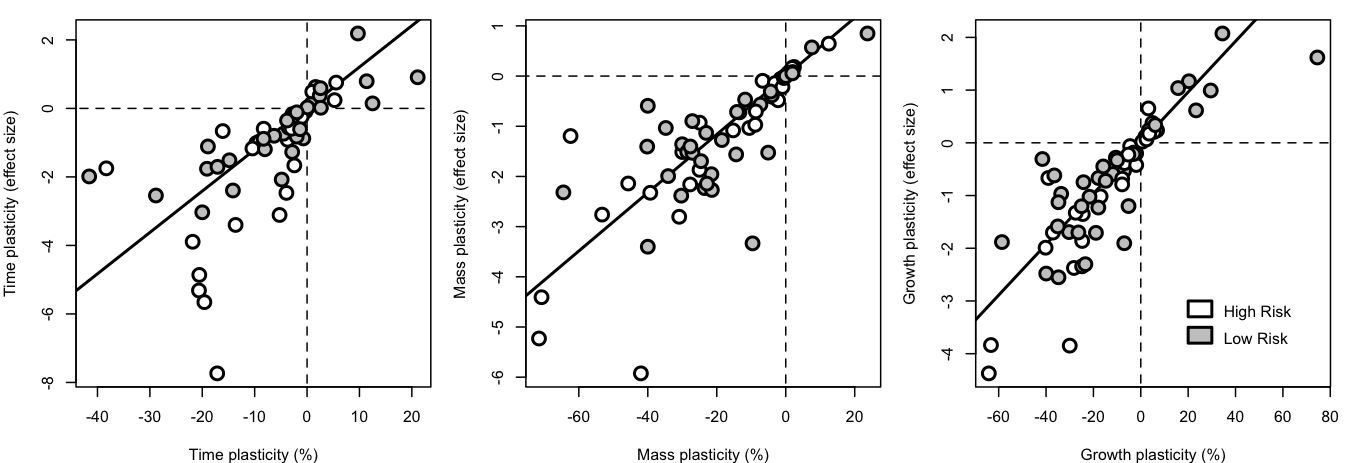


Figure S3. Phylogenetic hypothesis and phenotypic plasticity (the phylogeny in the left depicts the species codes listed in Table S1).


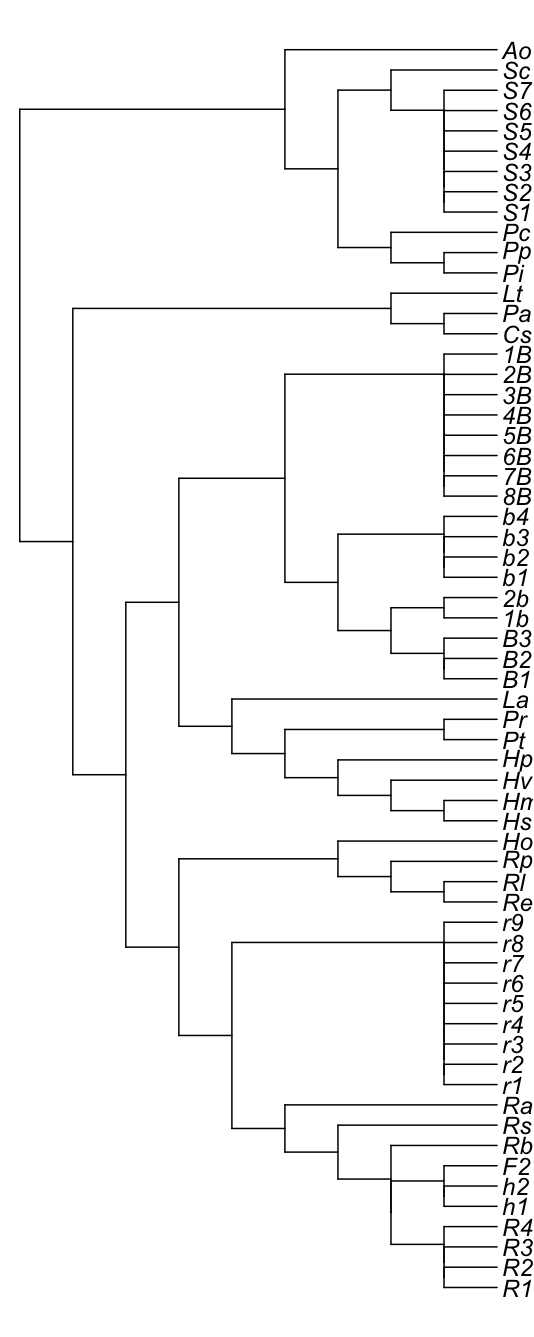

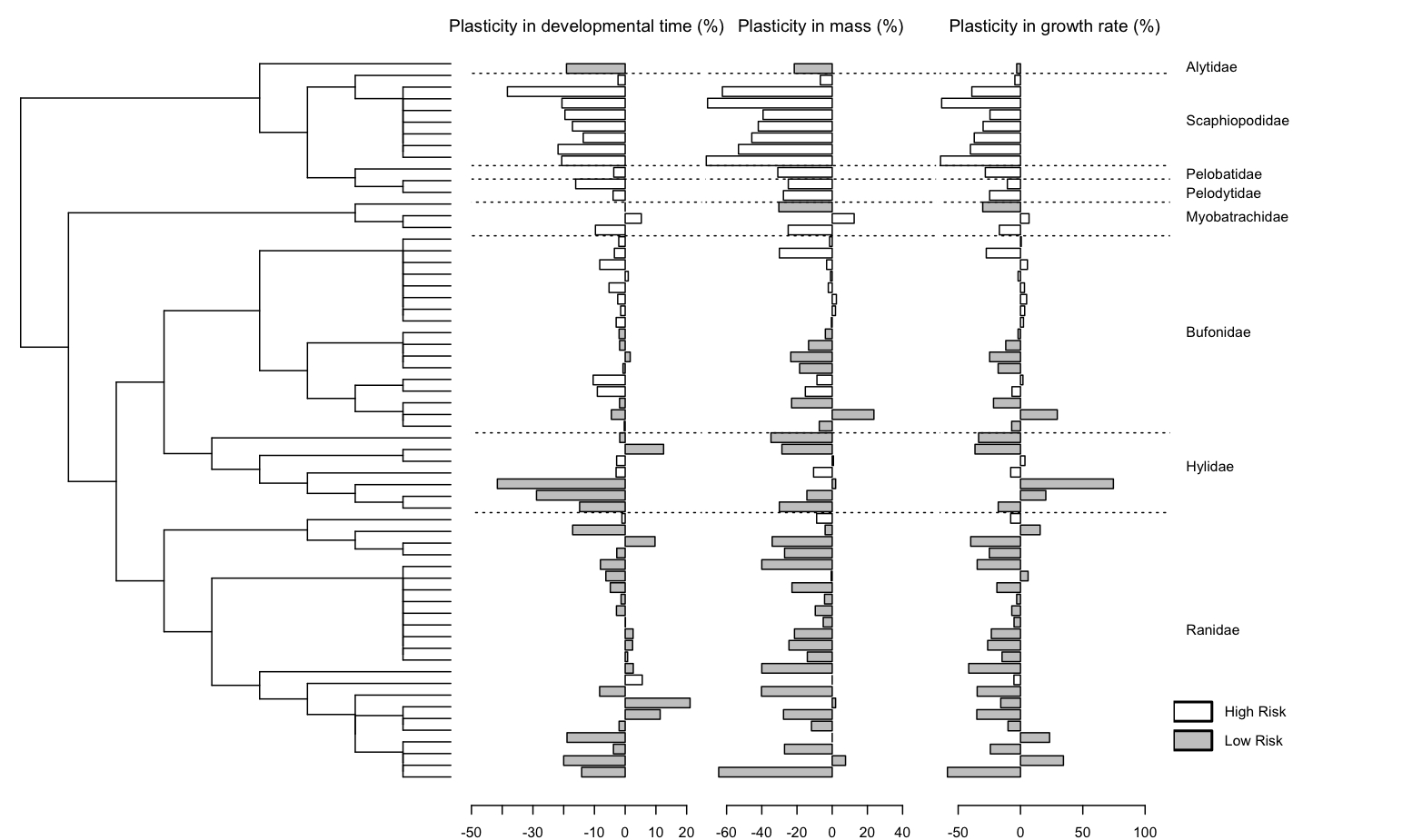


References

Adams, M.J. 2000. Pond permanence and the effects of exotic vertebrates on anurans. Ecological Applications 10: 559-568.

Blaustein, L., J. E. Garb, D. Shebitz, and E. Nevo. 1999. Microclimate, developmental plasticity and community structure in artificial temporary pools. Hydrobiologia 392:187–196.

Boone, M. D., E.E. Little, and R.D. Semlitsch. 2004. Overwintered bullfrog tadpoles negatively affect salamanders and anurans in native amphibian communities. Copeia 2004:683-690.

Brady, L.D. and R.A. Griffiths. 2000. Developmental responses to pond desiccation in tadpoles of the British anuran amphibians (*Bufo bufo*, *B. calamita* and *Rana temporaria*). Journal of Zoology 252:61-69.

Crump, M. L. 1989. Effect of habitat drying on development time and size at metamorphosis in *Hyla pseudopuma*. Copeia 1989:794-797.

Denver, R. J., N. Mirhadi, and M. Phillips. 1998. Adaptive plasticity in amphibian metamorphosis: Response of *Scaphiopus hammondii* tadpoles to habitat desiccation. Ecology 79:1859-1872.

Durnin, K. B. and G.R. Smith. 2001. Effects of changing water volume on the tadpoles of two anuran species (*Pseudacris triseriata* and *Rana blairi*). Journal of Freshwater Ecology 16:411-413.

Hamer, A.J., S.J. Lane, and M.J. Mahony. 2002. The role of introduced mosquitofish (*Gambusia holbrooki*) in excluding the native green and golden bell frog (*Litoria aurea*) from original habitats in south-eastern Australia. Oecologia 132:445–452.

Harris, R.N. 1999. The anuran tadpole: evolution and maintenance. In: *Tadpoles: the biology of anuran larvae.* (R.W. McDiarmid & R. Altig, eds), pp. 279-294. University of Chicago Press, Chicago.

Kendall, M., and A. Stuart. 1977. Advanced theory of statistics. London: Charles Grifﬁn & Company.

Kiesecker, J.M., and D.K. Skelly. 2001. Effects of disease and pond drying on gray tree frog growth, development, and survival. Ecology 82: 1956–1963.

Lane, S.J., and M.J. Mahony. 2002. Larval anurans with synchronous and asynchronous development periods: contrasting responses to water reduction and predator presence. Journal of Animal Ecology 71: 780–792.

Laurila, A. and J. Kujasalo. 1999. Habitat duration, predation risk and phenotypic plasticity in common frog (*Rana temporaria*) tadpoles. Journal of Animal Ecology 68:1123–1132.

Laurila, A., S. Karttunen, and J. Merila. 2002. Adaptive phenotypic plasticity and genetics of larval life histories in two *Rana temporaria* populations. Evolution 56:617–627.

Loman, J. 1999. Early metamorphosis in common frog *Rana temporaria* tadpoles at risk of drying: An experimental demonstration. Amphibia-Reptilia 20:421–430.

Morey, S., and D. Reznick. 2004. The relationship between habitat permanence and larval development in California spadefoot toads: field and laboratory comparisons of developmental plasticity. Oikos 104:172–190.

Pakkasma, S., and A. Laurila. 2004. Are the effects of kinship modified by environmental conditions in Rana temporaria tadpoles? Annales Zoologici Fennici 41:413-420.

Parris, M. J. 2000. Experimental analysis of hybridization in leopard frogs (anura: Ranidae): Larval performance in desiccating environments. Copeia 2000:11–19.

Pearman, P.B. 1993. Effects of habitat size on tadpole populations. Ecology 74:1982-1991.

Reques, R. and M. Tejedo. 1997. Reaction norms for metamorphic traits in natterjack toads to larval density and pond duration. Journal of Evolutionary Biology 10: 829–851.

Richter-Boix, A., G.A. Llorente and A. Montori. 2006. A comparative analysis of the adaptive developmental hypothesis in six Mediterranean anuran species along a pond permanency gradient. Evolutionary Ecology Research 8:1139-1154.

Rosenberg, M.S., D.C. Adams, and J. Gurevitch. 2000. MetaWin: statistical software for meta-analysis. Version 2.1. Sinauer Associates, Inc. Massachusetts, USA.

Rowe, C. L., and W.A. Dunson. 1995. Impacts of hydroperiod on growth and survival of larval amphibians in temporary ponds of central Pennsylvania, USA. Oecologia 102:397–403.

Ryan, T.J., and C.T. Winne. 2001 Effects of hydroperiod on metamorphosis in *Rana sphenocephala*. American Midland Naturalist 145:46–53.

Semlitsch R.D., and H.U. Reyer. 1992. Performance of tadpoles from the hybridogenetic *Rana esculenta* complex: interactions with pond drying and interspecific competition. Evolution 46:665–676.

Spieler, M. 2000. Developmental plasticity and behavioural adaptations of two West African anurans living in an unpredictable environment (amphibia, anura). Bonn Zoological Monographs 46:109–120.

Tejedo, M., and R. Reques. 1994. Plasticity in metamorphic traits of natterjack tadpoles: the interactive effects of density and pond duration. Oikos 71: 295-304.

Thum, K., and M. Mahoney. 2006. The effect of water level reduction on larval duration in the red-crowned toadlet *Pseudophryne australis* (Anura: Myobatrachidae): Bet-hedging or predictive plasticity? Amphibia-Reptilia 27:11-18.
